# Feline Tooth Resorption: A Description of the Severity of the Disease in Regard to Animal’s Age, Sex, Breed and Clinical Presentation

**DOI:** 10.3390/ani13152500

**Published:** 2023-08-03

**Authors:** Patrycja Pistor, Izabela Janus, Maciej Janeczek, Maciej Dobrzyński

**Affiliations:** 1 Department of Biostructure and Animal Physiology, Wroclaw University of Environmental and Life Sciences, Kożuchowska Str. 1/3, 51-631 Wrocław, Poland; 2 Department of Pathology, Wroclaw University of Environmental and Life Sciences, CK Norwida Str. 31, 50-375 Wrocław, Poland; izabela.janus@upwr.edu.pl; 3 Veterinary Dentistry and Orthodontics Centre, Osadnicza Str. 12, 51-515 Wroclaw, Poland; 4 Department of Pediatric Dentistry and Preclinical Dentistry, Wroclaw Medical University, Krakowska Str. 26, 50-425 Wrocław, Poland; maciej.dobrzynski@umw.edu.pl

**Keywords:** feline tooth resorption, feline odontoclastic resorptive lesion, TR, FORL, gingivitis, dental plaque, dental radiography

## Abstract

**Simple Summary:**

Oral diseases, including dental problems, are of great significance in domestic animals. Disorders involving tissue localised in the teeth, periodontium, gums or tongue can be associated with pain and loss of appetite. It is particularly prominent in predatory animals such as cats. One of the most prominent dental diseases in that animal species is tooth resorption (also known as odontoclastic resorptive lesion). The disease is associated with damage of the tooth tissue that eventually leads to tooth loss. We have analysed dental charts of 174 cats diagnosed with tooth resorption. The changes were most often noted in premolar and molar teeth. We have not found correlations between the severity of the disease and clinical symptoms shown by the animals, but the disease progressed with animals’ age. Based on the obtained results, we indicated the need of a careful dental examination with intraoral radiography in cats and especially in animals showing any signs of oral disease, even in cases with preserved appetite.

**Abstract:**

Feline tooth resorption (odontoclastic resorptive lesion) is a common and important issue in veterinary dentistry. This study aimed to analyse the disease’s severity and correlation with clinical information in the population of feline patients in Poland in the area of Lower Silesia. An analysis of the clinical charts of 174 cats with dental problems, which were diagnosed as tooth resorption, was conducted. The gender and breed had no influence on the disease severity, but the disease progressed with age. The lesions were mostly encountered within the third and fourth maxillary premolars (107, 108, 207, 208) and mandibular molars (309, 409). No direct correlation was found between the presence or severity of the disease and the clinical signs of affected cats. The study shows that feline tooth resorption is a common issue in feline dentistry and should be taken into account in all cases of animals with any signs of oral disease, including gingivitis and/or dental plaque with preserved appetite. A careful intraoral radiographic examination is essential to avoid false negative results in ambiguous cases.

## 1. Introduction

Feline oral diseases are considered to be a significant topic in veterinary medicine. The most frequent diseases in that group include: tooth resorption (TR; formerly feline odontoclastic resorptive lesion—FORL), lympho-plasmocytic (eosinophilic) stomatitis and periodontitis [1,2,3,4]. Tooth resorption was first described in cats as early as 1920 [5]. The first evidence of a TR-like disease comes from paleopathological descriptions of cats living 800 years ago [6]. However, paleopathological findings are rare, suggesting that TR is more of a modern problem. Similar changes to those occurring in the course of TR were described in 1930 by Hopewell-Smith and named odontolysis [7]. The report also describes histological changes present in the teeth. Moreover, Hopewell-Smith noted that the changes were associated with chronic marginal gingivitis. [7]. In the 1950s and 1960s, the disease was thought to be related to caries, and it was not until the 1970s that it was unequivocally determined that changes involved not caries, but resorption [8,9]. Tooth resorption has also been described in wild felids [10,11]. 

Due to the fact that TR can be accompanied by appetite loss and developing cachexia, a rapid and appropriate diagnosis as well as proper treatment are crucial for the animal’s wellbeing. The disease is characterised by a resorption in the cementum layer or enamel-covered surface of the tooth caused by polynuclear cells called odontoclasts. The lesions develop initially in the tooth ligament structures and cementum. As a result of the loss of root substance, the tooth destruction disturbs the stability of the crown causing its fracture [2,4,12]. Based on clinical examination and radiography, five stages of the disease have been distinguished [4]:

1. The resorption has minimal character. It involves the surface of the enamel or cementum with no loss of the dentine; the changes can be difficult to notice due to the low extent.
2. In this stage, the dentine loss is present, although the lesion does not involve dental pulp. The lesion has a mild character.
3. In the third stage of the disease, the resorption involves cementum, enamel, dentine and pulp cavity, but the tooth maintains its stability within the alveolar sac.
4. In the next stage, the resorption progresses involving all tooth structures and causes the loss of teeth stability.
5. The fifth stage can be subdivided into two types: 5a in which the tooth loses its crown while the root(s) remain unchanged apart from being significantly resorbed, and 5b in which a vast resorption of the root(s) is accompanied by the presence of an almost intact crown.

The division of either the fourth or fifth stage of the disease into further types differs between the authors. The American Veterinary Dental College suggests dividing the fourth stage into 4a, 4b and 4c types (https://avdc.org/; online access: 1 June 2023).

The disease aetiology is not understood completely, although many theories have been formed to explain this phenomenon. Factors that are being considered involve: the domestication of the cat (influencing the nutrition, vaccinations and neutering programs), as well as infectious agents and chronic inflammation associated with plaque accumulation. The contribution of anatomical factors related to the specific structure of feline teeth cannot be ruled out.

It is conceivable that the feline diet plays a role in the aetiology of TR [13,14]. The process of overfeeding with raw liver can contribute to the disease because both retinol and tretinoin present in the liver can directly stimulate the activity of clastic cells [4]. Moreover, cats with TR show significantly higher urine specific gravity and higher concentration of serum 25-hydroxyvitamin D3 (25(OH)D_3_) than cats without the disease, which confirms that the activity of odontoclasts is a result of not only local changes but also systemic disturbances [6]. Since food is the only source of vitamin D in cats, the dietary factor is decisive for the level of 25(OH)D_3_ in the blood serum. Taking the above-mentioned hypotheses into account, one cannot rule out that the domestication process and the resulting changes in feline diet play a significant role in the occurrence of TR. Nonetheless, finding the disease in wild cats casts doubt on this theory [14,15]. 

The role of viral infections in the course of the disease is poorly known. Some researchers describe the influence of calicivirus (FCV) on the development of TR [16]. Feline immunodeficiency virus (FIV) was also found in affected cats [17]. Significant metabolic changes were also localised in gingiva-derived stromal cells obtained from cats with TR and concomitant caliciviral infections [18]. However, the association of feline leukaemia virus (FeLV) with feline oral disease has not been demonstrated [19].

Chronic periodontitis has also been believed to have an influence on the development of odontoclastic lesions. The accumulation of dental plaque can cause inflammation of the periodontium t hat results in a local immune response and the release of inflammatory mediators (e.g., cytokines) and bacterial toxins (e.g., lipopolysaccharides), which play a significant role both in the stimulation of the inflammatory process and in the differentiation and infiltration of the clastic cells. Among the bacteria that may contribute to TR, gram negative anaerobic *Fusobacterium* and *Bacteroides* spp. [20] have been mentioned. In humans, antibodies against *Actinobacterium* sp. have also been found in the course of the resorptive disease. Although the majority of TR cases are combined with the presence of inflammation in the surrounding tissue, early lesions combined with a seemingly normal appearance of the tissue may cause confusion. Therefore, the inflammation is rather secondary than primary to the disease [6].

The specific structure of cats’ teeth may also contribute to the occurrence of TR. A lack of coverage of the dentin by cement in the area of the cementoenamel junction forming an area of increased root risk can be defined as a significant factor. The lack of cement and the presence of mineralized dentine could attract the odontoclast. A thin layer of cement in the area of furcation between the roots is another risk point [4].

It is also believed that mechanical forces and occlusal stress can contribute to the progression of TR. The repeating compressive and tensile forces caused by micro-movements during chewing and malocclusion can disrupt the chemical bonds between enamel rods, causing enamel abfraction and dentin exposure. Nonetheless, the disease can also affect teeth which are not subject to the abovementioned forces [4,6].

The disease incidence grows with age. Patients with tooth loss found during clinical examination are more susceptible to development of the disease [6]. The data regarding the incidence of TR are various, depending on diagnostic criteria. Some of the research is based on the results of clinical examination, while others also include the results of radiographic examination [3,21,22]. The early stages of the disease are very often not manifested by the animals and are difficult to discover during clinical examination. However, they can be recognised during radiography [21]. The affected teeth show gingivitis and often gingival hypertrophy. Cavities in dental crowns can be filled by gingival granulation tissue [23]. 

Veterinary medicine is gradually changing: on the one hand, diagnostic tools are constantly improving and becoming more available in routine practice; on the other hand, the awareness of owners has grown, leading to more careful care and providing more information to the veterinarian. Therefore, studies including animals’ history, clinical examination and specific procedures can improve our knowledge on changes in the disease prevalence and clinical picture. This study aimed at analysing: (1) the severity of the disease in affected animals based on radiographic examination and (2) the relationship between the disease severity and clinical signs noted by owners and during clinical examination.

## 2. Materials and Methods

The research included a retrospective analysis of data sheets of 174 cats presented for the first time to the Dental Clinic and diagnosed with tooth resorption. The group comprised 58% of males and 42% of females aged from 18 months to 18 years (median 7.5 years) representing various breeds with the majority (79%) representing domestic shorthair cats (Table 1).

All cats were diagnosed on the basis of the history, clinical examination and intraoral radiographic examination.

The medical history, including symptoms of oral disease, i.e., diminished or lost appetite, signs of pain and fetor ex ore was collected. All the animals underwent clinical examination. The presence of gingivitis, dental plaque and gingival bleeding (resulting from examination) were evaluated. The oral examination was performed initially on unanaesthetised animals and repeated after sedation for confirmation. The data obtained from examination without sedation were taken into account in the analysis. The clinical examination was complemented with an intraoral radiographic examination using iM3 Revolution 4DC device (iM3, Ireland) and CR 7 Vet Image Plate X-ray Scanner (iM3, Ireland). Radiological diagnostics were performed under general anaesthesia appropriate for each patients’ condition.

The number of absent teeth and affected teeth as well as the advancement of lesions were assessed on the obtained images. The severity of the disease was evaluated using staging available on the American Veterinary Dental College website (https://avdc.org/; online access: 1 June 2023).

The data underwent statistical analysis using StatisticaPL 13.3 (StatSoft, Kraków, Poland) software. The data normality were tested using Shapiro–Wilk analysis. The correlation of the results was tested using Spearman’s rang correlation analysis. The difference among groups was tested using either Mann–Whitney U analysis or Kruskal–Wallis analysis. The significance level was set at *p* < 0.05.

## 3. Results

**Figure 1:**
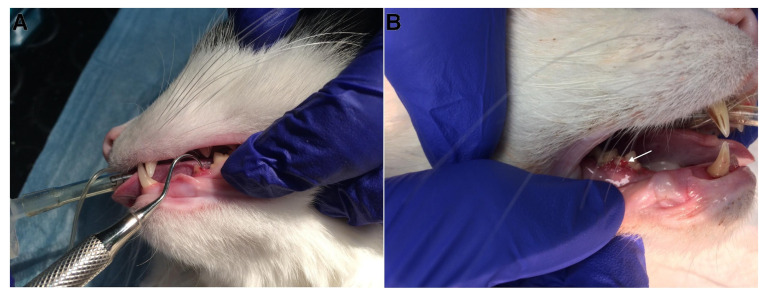
Clinical presentation of examined cats. Deep odontoclastic lesions penetrating to pulp cavity in examined teeth. (**A**) The lesion is located on the caudal aspect of 307 tooth; (**B**) the lesion is located on the buccal surface of 408 tooth (arrow), the 407 tooth is missing.

**Figure 2:**
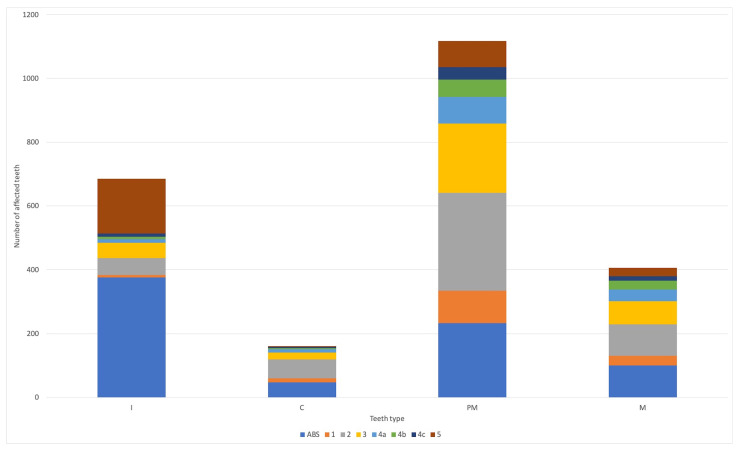
The degree of radiological lesions among individual teeth types in studied animals. ABS—absent; I—incisors; C—canine; PM—premolars; M—molars; 1–5—the degree of dental lesions according to American Veterinary Dental College (https://avdc.org/; online access: 1 June 2023).

**Figure 3:**
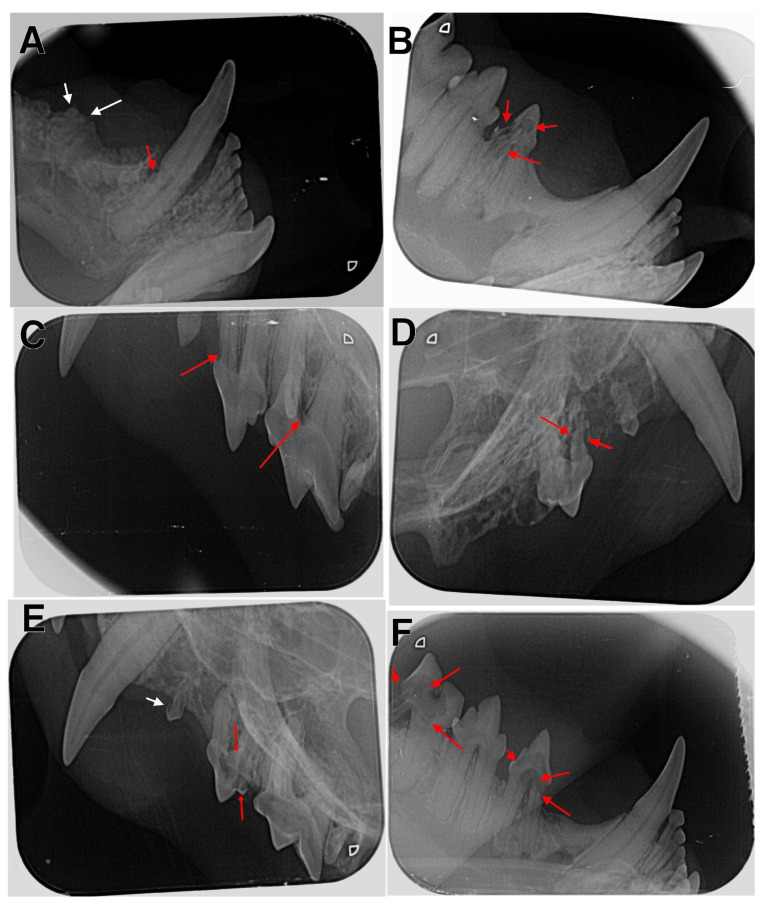
Radiographic examination of affected cats. (**A**) The right mandible with type 2 lesion in tooth 404 (red arrow) and type 5a lesion in tooth 407 (white arrows); (**B**) the right mandible with type 4 lesion in tooth 407; (**C**) the left maxilla with type 1 lesions in teeth 207 and 208 (red arrows); (**D**) the right maxilla with type 4 lesions in tooth107 (red arrows); (**E**) the left maxilla with type 1 lesion in tooth 206 (white arrow) and type 4 lesions in tooth 207 (red arrows); (**F**) the right mandible with type 4 lesions in teeth 407 and 409 (red arrows).

The radiographic examination of affected cats revealed lesions in 1613 teeth, inclusively. The pathological process was present in all types of teeth (from incisors to molars) and in all degrees of disease advancement (Figure 1, Figure 2 and Figure 3).

**Figure 4:**
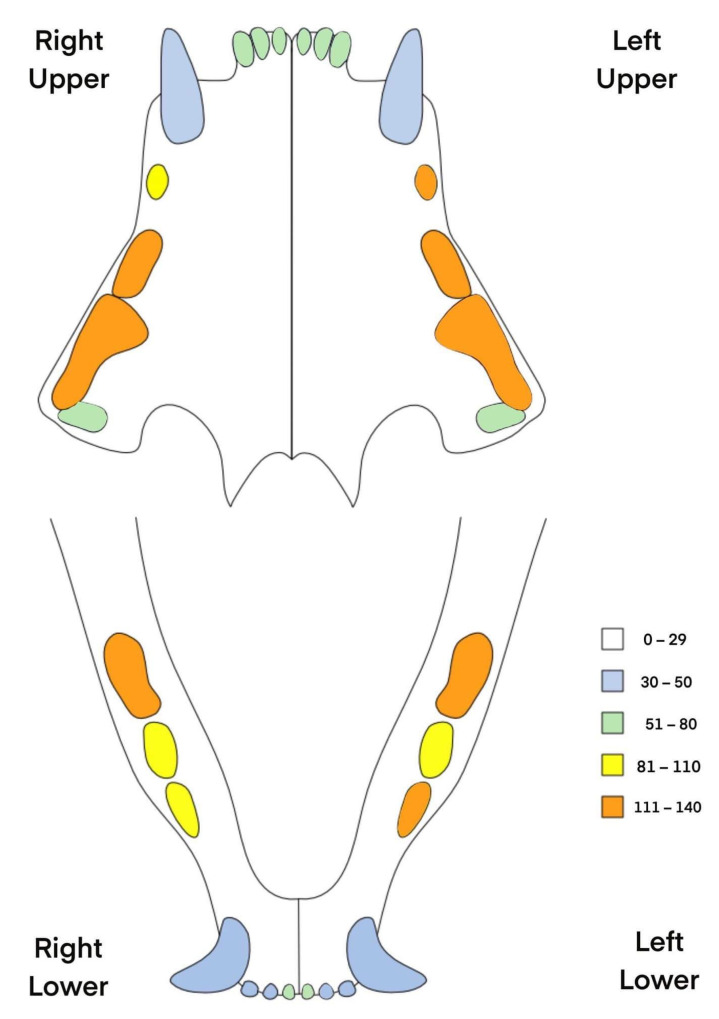
The incidence of resorptive lesion in each feline tooth in the examined animals. The most frequently affected teeth were maxillary premolars (107, 108, 206, 207, 208) and mandibular premolar and molars (307, 309, 409).

The examined cats showed resorptive lesions of 1–28 teeth (median 9 teeth). The lesions were noted in one (n = 9; 5.2%), two (n = 24; 13.8%), three (n = 36; 20.7%) or even four (n = 105; 60.3%) dental arches (Table 2). Among the 24 animals that showed lesions in two dental arches, the disease involved mainly only mandibles (n = 10) or maxilla (n = 9), followed by cats with the disease present only on the left side (n = 2) or present diagonally (n = 3). The lesions were mostly encountered within the third and fourth maxillary premolars (107, 108, 207, 208) and mandibular molars (309, 409) (Table 3; Figure 4). The affected teeth showed lesions scored from the first stage to the fifth stage with a median of the fifth stage.

Moreover, a total number of 756 teeth were missing (Table 3) in the examined animals with a medium value of 3 per animal and a maximum value of 20 in one animal. The most commonly missing teeth included: 106 (n = 62), 206 (n = 53), 301 (n = 46) and 401 (n = 46).

There was a correlation between the animals’ age and number of missing teeth (*p* < 0.05; r = 0.37; Spearman correlation analysis), number of affected teeth (*p* < 0.05; r = 0.19; Spearman correlation analysis) and the highest degree of lesions (*p* < 0.05; r = 0.29; Spearman correlation analysis). Moreover, we have noted a positive correlation (Spearman correlation analysis) between the degree of lesions and the animal’s age in teeth number: 107 (*p* < 0.05; r = 0.23), 108 (*p* < 0.05; r = 0.22), 208 (*p* < 0.05; r = 0.29), 307 (*p* < 0.05; r = 0.38), 404 (*p* < 0.05; r = 0.53) and 409 (*p* < 0.05; r = 0.30). We have also noted multiple correlations in the degree of observed lesions between corresponding groups of teeth (Table 4 and Table 5).

There was no significant difference between males and females regarding the number of affected teeth, number of missing teeth and the highest degree of lesions (*p* > 0.05; Mann–Whitney U analysis); also, there was no difference in the above mentioned parameters in relation to animals’ breed (*p* > 0.05; Kruskal–Wallis analysis).

A diminished appetite was noted in 37 (21.3%) cats, fetor ex ore in 14 (8%) cats, the presence of gingivitis in 89 (51.1%), dental plaque in 67 (38.5%) animals and gingival bleeding during examination in 24 (13.8%) animals. Apart from gingivitis, no other signs of inflammation were noted (including faucitis). There were no differences in the number of affected teeth or number of affected dental arches depending on the presence of clinical symptoms (*p* > 0.05).

## 4. Discussion

Diseases involving the lysis of dental hard tissues (including TR) resulting from the activity of blast cells were described in various animal species as well as in humans [24,25,26]. In horses, a disease resembling TR was reported for the first time as late as 2008 [27]. Tooth resorption is less common in dogs than in cats. In the case of dogs, we more often deal with external replacement resorption and external inflammatory resorption [28]. As in the case of cats, the cause of the disease cannot be clearly identified [29]. The cause of the disease differs among species. In humans and cats, the lysis predominates being accompanied partly by inflammation and very limited hypercementosis, while in horses, a very intense periodontitis combined with secondary hypercementosis dominates [25,30,31,32]. In horses, the disease affects mostly incisors and canine teeth, whereas in cats the lesions are most frequently present in premolar and molar teeth [3,25,33]. Ingham et al. in a study of 228 cats found that teeth 307 and 407 were most commonly affected, with the lesions being symmetrical in most cases [3]. Also in our study, the cheek teeth were predominantly affected but teeth number 107, 108, 207, 208, 309 and 409 were more frequently affected than teeth number 307 and 407. Although Ingham et al. [3] showed that the disease was most frequently affecting the mandibular teeth, the majority of cats presented in our study showed changes in three or all four dental arches. Moreover, we have shown that teeth of the same type (canine or shearing teeth) or of the ipsilateral (right, left, mandibular, maxillar) tend to have a similar severity of changes. It points to the need for a complete oral examination and a full oral radiography in a case of noticing changes in at least one tooth.

The data regarding the incidence of TR in cats are diverse, which is likely caused by the differences in diagnostic processes [3,21]. The number of publications on this disease in cats is not wide. Our study consisted of clinical history, clinical examination and intraoral radiographic examination. The number of patients is large and allows conclusions to be drawn. The study conducted by Ingham et al. [3] on 228 domestic shorthair cats and 9 British shorthair cats showed TR in 29% of animals, with females being affected more often than males. Lund et al. [34] noted TR in 48% of cats older than one year. In our study, we have recognised TR with no influence of sex or breed on the presence of the disease, but with more advanced disease in older animals (higher number of affected and/or missing teeth and higher degree of changes).

It seems that since TR’s first descriptions in the 1920’s, the disease’s incidence has increased. In addition to the improvement in diagnostic possibilities, it may be related to other factors, e.g., animal nutrition [4]. What is riveting is a low percentage of animals showing loss of appetite despite the severity of the lesions. This may be related to the fact that cats are strict carnivores which is reflected in the way they take and handle food. They show an increase in their teeth’s ability to cut and a concomitant loss of their ability to crush food, which also applies to the molars. Thus, the food taken in is subjected to little processing, apart from grinding it into smaller particles [35]. Hence, the involvement of the chewing apparatus when eating dry commercial feed is low [36]. Cats tend to swallow such food. On the other hand, due to disorders affecting part of the periodontal mechanoreceptors, they may disrupt the chewing process [37]. The most common clinical signs were gingivitis and presence of dental plaque, pointing to a conclusion that a thorough dental examination including intraoral radiography should be performed in all cats showing any signs of gingivitis and/or dental plaque, regardless of their appetite and dietary habits.

Unfortunately, due to a retrospective character of our study, the data regarding animals’ diet, vaccination history or viral infection status were not available, therefore could not serve for extended analysis.

The obtained results show explicitly that TR is a very common feline disease that requires intraoral radiographic examination as an addition to clinical examination of cats, especially when the results of clinical examination are ambiguous. It is consistent with the results presented by other authors [3,21]. Our results highlight the need of a through oral examination (including intraoral radiography) in any case of suspicion of oral disease or oral pain and moreover, in all elderly cats. Although previously third mandibular premolar teeth were considered the most frequently affected [3], we have shown that the disease can be present more often in other teeth (including maxillary teeth).

## 5. Conclusions

TR should be taken into account in the differential diagnosis in all cases of cats with appetite disorder, signs of oral pain, gingivitis, dental plaque and fetor ex ore, regardless of the animal’s age. The most affected teeth are premolar and molar teeth with a tendency to involve multiple teeth of the same group. An intraoral radiographic examination is essential to confirm the disease in ambiguous cases with nonspecific clinical symptoms.

## Figures and Tables

**Table 1:** The breed distribution in the examined group.

Animal’s Breed	Number
Domestic shorthair	138
British shorthair	9
Maine coon	9
Ragdoll	7
Persian	3
Siamese	2
Thai	2
Neva Masquarade	1
Peterbald	1
Devon Rex	1
Scottish longhair	1

**Table 2:** The number and arrangement of involved dental arches in examined animals.

Number and Arrangement of Affected Dental Arches		Number of Affected Animals
1 dental arch	left maxilla	4
	left mandible	4
	right mandible	1
2 dental arches	bilateral mandible	10
	bilateral maxilla	9
	only left side	2
	diagonally	3
3 dental arches		36
4 dental arches		105

**Table 3:** The table presents the number of animals showing tooth resorption of various degrees in each of the examined teeth, including information about missing teeth; ABS—absent.

Degree of Lesions/Number of Tooth	ABS	1	2	3	4a	4b	4c	5	SUM	SUM w/o ABS
101	29		4	2				34	69	40
102	23		4	6	3	3	2	17	58	35
103	32		6	7	2		1	14	62	30
104	17		10	3	2		1	1	34	17
106	62	4	16	15	2	3	3	5	110	48
107	20	12	31	28	10	9	5	9	124	104
108	14	8	33	36	13	8	3	3	118	104
109	31		18	14	9	1	1	3	77	46
201	32	1	3	6		2	3	27	74	42
202	24	3	4	1	1	1	1	21	56	32
203	25		9	8	3	1	2	12	60	35
204	6	4	15	4	2	1			32	26
206	53	3	27	15	3	1	2	7	111	58
207	14	16	48	19	11	5	5	2	120	106
208	15	19	31	31	10	4	2	3	115	100
209	36	1	10	9		1		5	62	26
301	46		2	2			1	11	62	16
302	34		3	2	1			8	48	14
303	26	1	6	6			1	4	44	18
304	13	3	20	5	3	3	1	1	49	36
307	20	8	21	15	9	9	8	24	114	94
308	5	11	35	31	10	3	2	5	102	97
309	16	15	35	25	14	13	10	6	134	118
401	46	1	5	3				6	61	15
402	32	1	2	4				9	48	16
403	27		5	1	2			8	43	16
404	11	6	14	10	3		1	1	46	35
407	26	7	25	9	9	6	8	18	108	82
408	4	13	40	18	7	6	1	6	95	91
409	17	15	35	24	14	13	2	13	133	116
SUM	756	152	517	359	143	93	66	283		

**Table 4:** Correlations in the severity of the disease (degree of tooth resorption) between teeth within the same type; Spearman correlation analysis.

Group of Teeth	Pair of Teeth	*p*-Value	r-Value
canine teeth	104–204	*p* < 0.05	0.79
	104–304	*p* > 0.05	
	104–404	*p* < 0.05	0.74
	204–304	*p* > 0.05	
	204–404	*p* < 0.05	0.67
	304–404	*p* < 0.05	0.45
shearing teeth	107–108	*p* < 0.05	0.33
	207–208	*p* < 0.05	0.37
	307–308	*p* < 0.05	0.47
	307–309	*p* < 0.05	0.26
	308–309	*p* < 0.05	0.51
	407–408	*p* < 0.05	0.37
	407–409	*p* < 0.05	0.33
	408–409	*p* < 0.05	0.36

**Table 5:** Correlations in the severity of the disease (degree of tooth resorption) between shearing teeth within right side, left side, maxilla or mandible; Spearman correlation analysis.

Group of Teeth	Pair of Teeth	*p*-Value	r-Value
right side	107–407	*p* < 0.05	0.41
	107–408	*p* < 0.05	0.26
	107–409	*p* < 0.05	0.29
	108–407	*p* < 0.05	0.28
	108–408	*p* > 0.05	
	108–409	*p* < 0.05	0.40
left side	207–307	*p* > 0.05	
	307–308	*p* > 0.05	
	307–309	*p* < 0.05	0.24
	208–307	*p* > 0.05	
	208–308	*p* > 0.05	
	208–309	*p* < 0.05	0.53
maxilla	107–207	*p* < 0.05	0.44
	107–208	*p* < 0.05	0.25
	108–207	*p* < 0.05	0.32
	108–208	*p* < 0.05	0.53
mandible	307–407	*p* < 0.05	0.43
	307–408	*p* > 0.05	
	307–409	*p* < 0.05	0.24
	308–407	*p* > 0.05	
	308–408	*p* < 0.05	0.31
	308–409	*p* < 0.05	0.54
	309–407	*p* > 0.05	
	309–408	*p* > 0.05	
	309–409	*p* < 0.05	0.49

## Data Availability

The data that support the findings of this study are available from the corresponding author, upon reasonable request.
